# The influence of digital PET/CT on diagnostic certainty and interrater reliability in [^68^Ga]Ga-PSMA-11 PET/CT for recurrent prostate cancer

**DOI:** 10.1007/s00330-021-07870-5

**Published:** 2021-04-15

**Authors:** Ian Alberts, Jan-Niklas Hünermund, Christos Sachpekidis, Clemens Mingels, Viktor Fech, Karl Peter Bohn, Axel Rominger, Ali Afshar-Oromieh

**Affiliations:** Department of Nuclear Medicine, Inselspital, Bern University Hospital, University of Bern, Freiburgstr. 18, 3010 Bern, Switzerland

**Keywords:** Prostate cancer, Positron emission tomography, Nuclear medicine, Molecular imaging

## Abstract

**Objective:**

To investigate the impact of digital PET/CT on diagnostic certainty, patient-based sensitivity and interrater reliability.

**Methods:**

Four physicians retrospectively evaluated two matched cohorts of patients undergoing [^68^Ga]Ga-PSMA-11 PET/CT on a digital (dPET/CT *n* = 65) or an analogue scanner (aPET/CT *n* = 65) for recurrent prostate cancer between 11/2018 and 03/2019. The number of equivocal and pathological lesions as well as the frequency of discrepant findings and the interrater reliability for the two scanners were compared.

**Results:**

dPET/CT detected more lesions than aPET/CT (*p* < 0.001). A higher number of pathological scans were observed for dPET/CT (83% vs. 57%, *p* < 0.001). The true-positive rate at follow-up was 100% for dPET/CT compared to 84% for aPET/CT (*p* < 0.001). The proportion of lesions rated as non-pathological as a total of all PSMA-avid lesions detected for dPET/CT was comparable to aPET/CT (61.8% vs. 57.0%, *p* = 0.99). Neither a higher rate of diagnostically uncertain lesions (11.5% dPET/CT vs. 13.7% aPET/CT, *p* = 0.95) nor discrepant scans (where one or more readers differed in opinion as to whether the scan is pathological) were observed (18% dPET/CT vs. 17% aPET/CT, *p* = 0.76). Interrater reliability for pathological lesions was excellent for both scanner types (Cronbach’s *α* = 0.923 dPET/CT; *α* = 0.948 aPET/CT) and interrater agreement was substantial for dPET/CT (Krippendorf’s *α* = 0.701) and almost perfect in aPET/CT (*α* = 0.802).

**Conclusions:**

A higher detection rate for pathological lesions for dPET/CT compared with aPET/CT in multiple readers was observed. This improved sensitivity was coupled with an improved true-positive rate and was not associated with increased diagnostic uncertainty, rate of non-specific lesions, or reduced interrater reliability.

**Key Points:**

*• New generation digital scanners detect more cancer lesions in men with prostate cancer.*

*• When using digital scanners, the doctors are able to diagnose prostate cancer lesions with better certainty*

*• When using digital scanners, the doctors do not disagree with each other more than with other scanner types*.

## Introduction

The introduction of the first commercially available combined PET/CT scanner in 2001 marked a significant milestone for nuclear medicine [1]. Traditionally, the first detectors were based on blocks of bismuth germinate (BGO) scintillation crystals coupled with photomultiplier tubes and such systems (“aPET/CT”) have inherent physical limits to their performance based on this analogue technology. The recent introduction of solid-state detection systems (commonly termed “digital” PET/CT, dPET/CT) marks a second important milestone [2]. Fully digital systems demonstrate a number of technical advantages, which include a better coupling between the crystal and photodetectors, increased spatial resolution and sensitivity, improved background-to-noise, faster time-of-flight (TOF), and associated advanced TOF reconstruction. In addition, the state-of-the-art digital systems often include longer axial coverage, smaller crystals, and more advanced electronics, which lead to higher sensitivity, higher spatial resolution, and shorter deadtime. The favourable performance characteristics of such fully digital systems have been confirmed by a number of publications [3], which correspond to improvements in image quality and lesion detection [4–6], including in PET/CT with [^68^Ga]Ga-PSMA-11 [7].

A number of publications report excellent diagnostic performance with PSMA tracers, with high sensitivity and specificity [2–5, 8–10]. One head-to-head study comparing dPET/CT with aPET/CT in 2-[^18^F]FDG PET/CT showed upstaging of patients (13%) and improved image quality [11]. Nguyen et al found increased image quality, standardised uptake value (SUV), and lesion sharpness. They also identified additional lesions in dPET/CT in their cohort of 21 patients in 2-[^18^F]FDG for oncological patients [4]. Improved detection rates in dPET/CT with [^68^Ga]Ga-PSMA-11 have been found, particularly at low PSA values [7]. However, we find no publications that consider the influence of dPET/CT on diagnostic certainty and interrater reliability. Lopez-Mora et al report agreement in [^18^F]choline dPET/CT for parathyroid imaging; however, only the agreement between dPET/CT and aPET/CT was reported, rather than interrater reliability [5].

Likewise, a number of publications report high interrater reliability for imaging with PSMA radioligands [12]. Although PC lesions exhibit exquisite overexpression of PSMA [13], unfortunately, this is not uniquely so. PSMA is expressed both in the neovasculature of other solid tumours [14], physiologically in tissues such as ganglia which represent potential pitfalls [15–18] and mediastinal and thoracic lymph nodes [19]. False positives, such as rib fractures [20], and false negatives, such as PSMA-non avid metastases, have been reported [21, 22]. Even at very high prostate-specific antigen (PSA) values, roughly 5% of PSMA-PET/CT scans are negative due to PSMA-negative disease, possibly as a result of tumour dedifferentiation [23, 24]. Both anecdotal evidence and a retrospective cohort study confirm increased rates of non-specific tracer uptake in new generation tracers such as [^18^F]PSMA-1007 when compared with [^68^Ga]Ga-PSMA-11 [25]. Case reports of diagnostically uncertain lesions in PSMA-PET abound in the literature, which can result in unnecessary and invasive examinations [26]; diagnostic confidence with PSMA radioligands is therefore of high clinical importance. Whereas a number of studies report only binary scales (pathological vs. benign), diagnostically equivocal or indeterminate lesions are clinically relevant. For example, Yin et al report a follow-up study of 56 patients with indeterminate findings (using a 5-point PSMA-RADS scale), with 48% of lesions at follow-up imaging showing changes indicative of PC [27].

Initial studies with dPET/CT report increased diagnostic performance as an advantage of digital versus analogue systems [4]. The aim of this study is to investigate the effect of dPET/CT on diagnostic certainty and interrater reliability in PET/CT with [^68^Ga]Ga-PSMA-11 in biochemically recurrent PC.

## Materials and methods

### Patient population

In this retrospective analysis, we included 65 consecutive individuals who were examined on our digital PET/CT (dPET/CT) between 10/2018 and 03/2019. Sixty-five corresponding patients examined on one of two cross-calibrated analogue PET/CT scanners (aPET/CT) were included, with PSA; age; T, N, and M stage (TNM, 8^th^ edition); and Gleason score (GS) as closely matched as possible. For the aPET/CT, only patients examined prior to the installation of our dPET/CT in 10/2018 were included (11/2018-03/2019), to remove the choice of scanner as a potential source of bias.

Clinical characteristics for our cohorts are outlined in Table 1. All patients were referred to our centre for [^68^Ga]Ga-PSMA-11 PET/CT in the setting of biochemically recurrent PC. All patients in each group underwent initial treatment either radical prostatectomy alone (*n* = 55 per cohort) or combined radical prostatectomy and radiotherapy (*n =*10). Patients undergoing androgen deprivation therapy (ADT) in the previous 6 months were excluded [28]. No significant differences in age, GS, or PSA value were observed between the two cohorts (*p* > 0.05). This study was performed in accordance with the Helsinki Declaration and was approved by the regional ethics commission (KEK 2018-00299) where informed consent for retrospective analysis of patient data was waived.

**Table 1:** Matched-pair cohort characteristics: prior treatment operation (OP) or combined operation + radiotherapy (OP + RT). Clinical parameters: Gleason score (GS), age (years), PSA (ng/ml), and TNM stage (Union for International Cancer Control UICC, 8^th^ Ed.) No significant differences in age, GS, or PSA value were observed between the two cohorts (*p > *0.05)

Parameter	Digital	Analogue
Prior treatment	OP (*n* = 55); OP+RT (*n* = 10)	OP (*n* = 55); OP+RT (*n* =10)
Age (median, range)	68 (48 - 83)	68 (50 - 81)
T stage (median, range)	3 (1 - 4)	3 (1 - 3)
N stage (median, range)	0 (0 - 1)	0 (0 - 1)
M stage (median, range)	0 (0 - 1)	0 (0 - 1)
GS (median, range)	7 (6 - 9)	7 (6 - 9)
PSA value (mean ± SD)	9.03 ± 28.00	5.66 ± 11.41

### Radiotracer

[^68^Ga]Ga-PSMA-11 was produced as previously described [28, 29]. The radiopharmaceutical was given by intravenous bolus injection with a weight-adjusted target dose of 3MBq/kg. For the dPET/CT, the mean body-weight-adjusted dose was 219 ± 34 MBq (median 207, range 162-312 MBq), and for the aPET/CT, mean 199 ± 19 MBq (median 199, range 141-222 MBq).

### Imaging

Regular whole-body PET scans (from head to the thighs) were performed for all individuals at 1.5h p.i following oral hydration with 1 L of water (beginning from 30 min p.i.) and 20mg of i.v. furosemide (at 1 h p.i.). as per our previously published protocol [30].

### Image acquisition

All patients were investigated using either a Biograph-VISION 600 PET/CT digital scanner (*n* = 65) “dPET/CT” or one of two cross-calibrated Biograph-mCT PET/CT analogue scanners (*n* = 65) (“aPET/CT”) (both: Siemens). The examination protocols are as previously published [7].

### Image evaluation

Image analysis was performed using an appropriate workstation and software (SyngoVia; Siemens). Four physicians (one board-certified nuclear medicine physician and three residents of varying seniority, see supplementary materials) read all scans independently. Readers were blinded to patient demographics, clinical details, scan date, and scanner type when reviewing scans. Lesions were classified using previously published PSMA-RADS (1.0) criteria [31]. Prior to the study, all readers were provided with literature describing PSMA-RADS, visual criteria for the identification of pathological lesions, and known pitfalls [15, 17, 19].

Lesions were grouped as definitively benign/likely benign (PSMA-RADS 1-2), equivocal (PSMA-RADS 3A-3D), and pathological (i.e. likely cancer/definitively cancer (PSMA-RADS 4-5). To limit variation in lesion numbers in highly polymetastatic patients, a maximum of five lesions per category were recorded (the five most visually prominent lesions per category). A rate of non-specific findings was defined (= detected lesions classified as non-pathological or equivocal, i.e. PSMA-RADS 1-3 as a proportion of all lesions detected). A rate of equivocal findings (lesions classified as uncertain, i.e. PSMA-RADS 3A-3D as a proportion of all lesions detected) was defined as a measure of diagnostic uncertainty.

In the second step, scans where a majority of readers (≥ 3) were in agreement that at least one definite pathological lesion was present (PSMA-RADS 4-5) were recorded as “pathological” and those without pathological (PC) lesions as “negative” in a binary scale (= patient-based sensitivity) [32]. The number of scans where differences in opinion were noted was recorded as “discrepant”.

### Statistical analysis

Statistical analyses were performed using Excel (Microsoft) and SPSS (IBM). Comparisons for detection frequency for various lesion types (pathological, benign, and uncertain) at both aPET/CT and dPET/CT were made by Pearson’s chi-squared test.

*p* values < 0.05 were considered statistically significant. The rates of discrepant scan findings (using a binary scale) and true-positive rate were compared between scanner types by the binomial test. The frequency of findings by the reader was compared by the chi-squared test and rate of findings by the unpaired two-tailed Student’s *t* test. Cronbach’s *α* was calculated as a test of interrater reliability, interrater agreement was tested by Krippendorf’s *α* [33]. Interpretation of agreement was according to Landis and Koch [34]. Correlations were tested by Pearson’s pairwise correlation.

### Follow-up

Clinical follow-up for clinical confirmation or refutation of scans rated as pathological was performed for all patients (minimum 1 year of follow-up). Where available, PSA, subsequent treatment, and correlative imaging were collected. Validation criteria were used as previously published [9] and using standard reporting for diagnostic accuracy (STARD) guidelines [35]. Correlative imaging, biopsy, or fall in serum PSA following targeted radiotherapy of a lesion rated as pathological were considered confirmatory or refutation of a scan rated as pathological or negative on a patient-based level. Details are in Table 2, supplementary materials.

## Results

### Lesion detection rate

**Fig. 1:**
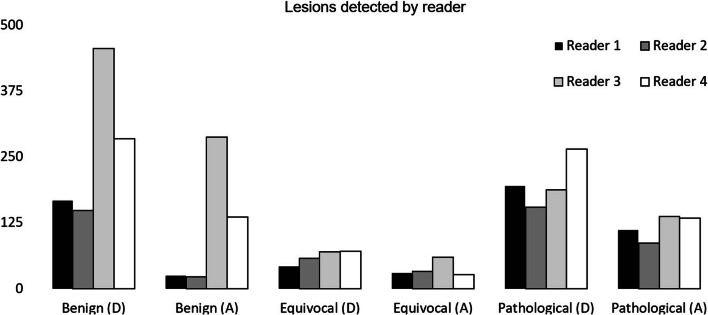
Total number of lesions identified by each reader across both scanners where (A) = aPET/CT and (D) = dPET/CT. Greater numbers of benign, equivocal, and pathological lesions were identified by all four readers combined at dPET/CT (*p* < 0.05)

All four readers combined detected a total of 3671 lesions in *n* = 130 patients. The results by lesion type (benign, equivocal, and pathological) and reader are shown in Fig. 1. For all individual readers, greater numbers of lesions were identified at dPET/CT compared to those at aPET/CT (*p* < 0.0001).

### Rate of non-specific and equivocal findings

**Fig. 2:**
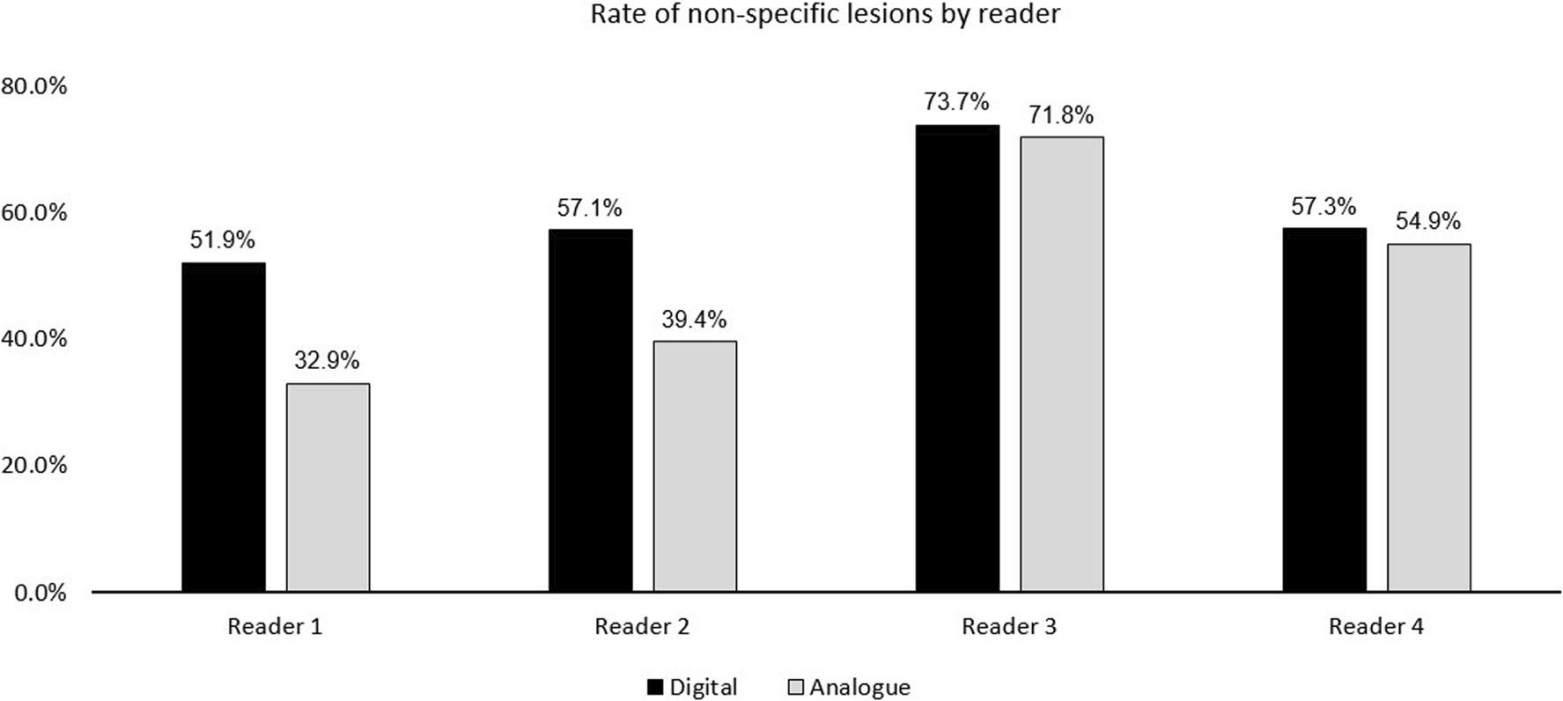
The rate of non-specific findings (NSF = detected lesions classified as non-pathological or equivocal, i.e. PSMA-RADS 1-3 as a proportion of all lesions detected)

The rate of non-specific findings is shown in Fig. 2. No significant difference was observed for dPET/CT compared to aPET/CT (mean rate for all readers 60.7% vs. 56.4%, *p* > 0.05). Differences in reader experience were noted, however. The two more experienced readers (readers 1-2) exhibited overall lower rates of non-specific findings compared to the two more junior readers (readers 3-4).

**Fig. 3:**
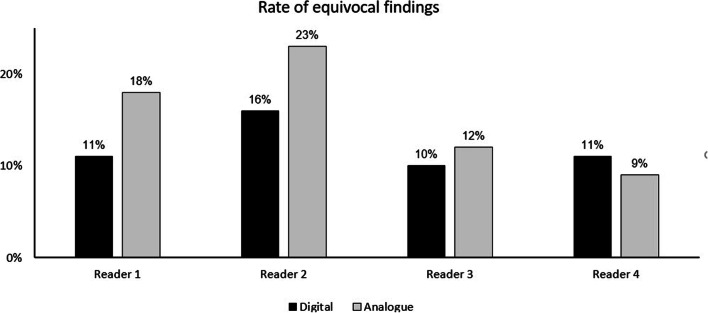
Rates of diagnostically uncertain lesions (equivocal) as a percentage of the total PSMA-avid lesions detected

The rate of equivocal (i.e. PSMA-RADS 3A-3D) findings per reader is shown in Fig. 3. Likewise, no statistically significant differences were identified for dPET/CT compared to those for aPET/CT (mean rate for all readers 11.7% vs. 12.6%, *p* > 0.05). Noteworthy is that while readers 1-3 all showed a lower rate of uncertain findings, the fourth (most junior) reader demonstrated a slightly higher, albeit non-significant rate at dPET/CT (11.4%) compared to that at aPET/CT (9.1%). To test for the hypothesis that uncertain lesions are at risk of misclassification, the correlation between the number of lesions classified as pathological and equivocal were compared, with no correlation being observed between the number of pathological and equivocal lesions for dPET/CT (*r* = 0.168) or for aPET/CT (*r* = 0.092).

#### Patient-based sensitivity

Scans with at least one pathological lesion (PSMA-RADS 4-5) were rated as “pathological” and those with zero pathological lesions were rated as “negative” for each reader, as described above. A significantly higher patient-based sensitivity (number of scans rated by all four readers as pathological) was observed for dPET/CT (83% vs. 57%, *p* < 0.0001). A post hoc power analysis confirms that the study was sufficiently powered to show this higher sensitivity with *n* = 130 participants with 90.8% power.

**Fig. 4:**
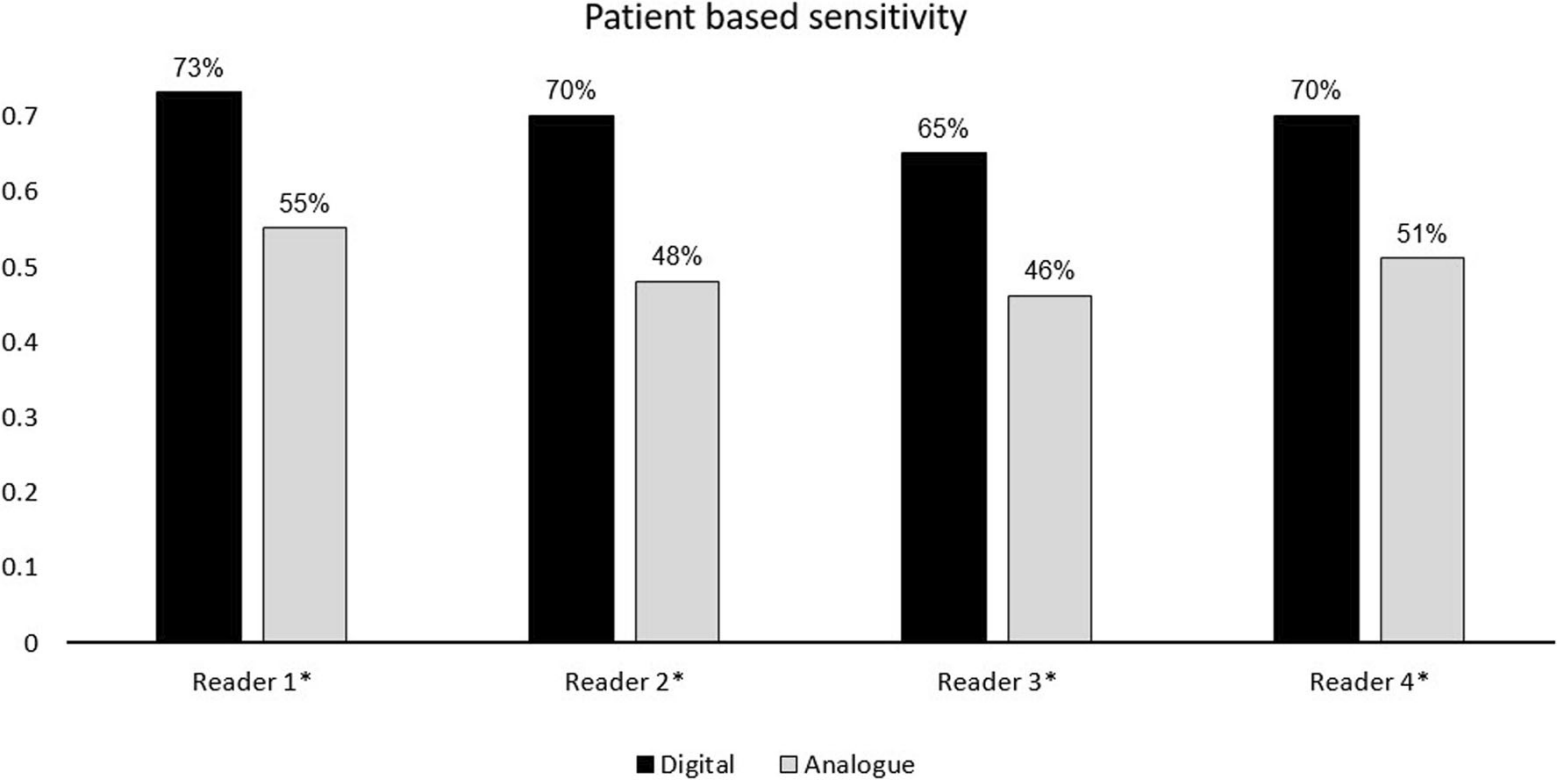
Patient-based sensitivity for all four readers (= scans rated as pathological, i.e. at least one PC lesion in PSMA-RADS category 4-5). Statistically significant results are marked with an asterisk (*)

All four readers demonstrated a higher rate patient-based sensitivity (= number of pathological pets) on dPET/CT compared with that on aPET/CT (reader 1, 73% vs. 55%; reader 2, 70% vs. 48%; reader 3, 65 vs. 46%; reader 4, 70% vs. 51%; *p < *0.05). The results are shown in Fig. 4.

### Clinical follow-up

**Fig. 5:**
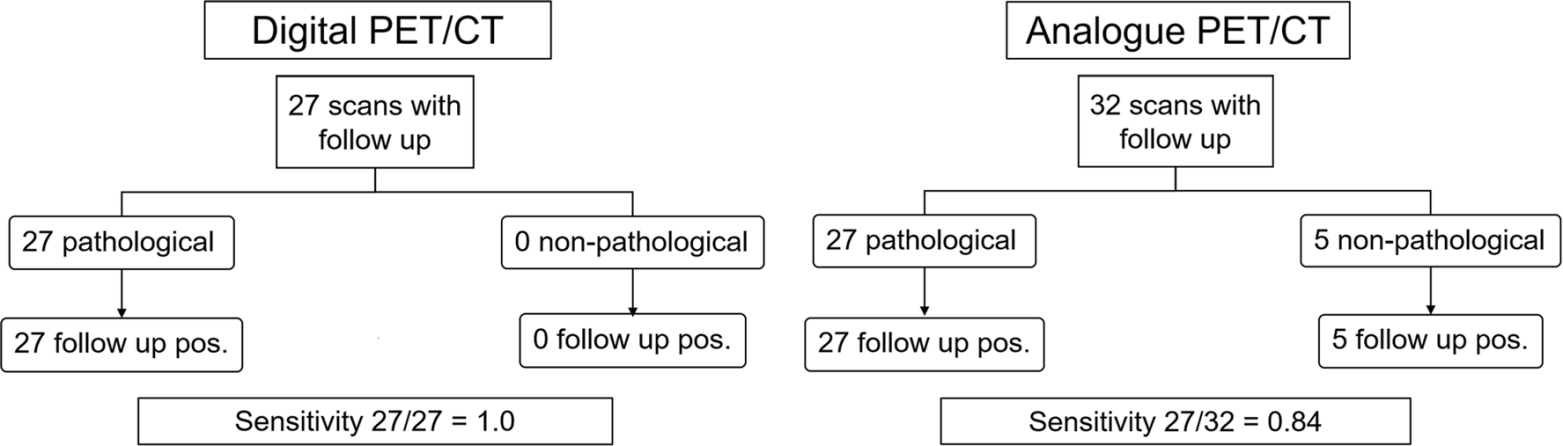
STARD flowchart showing the number of pathological scans where follow-up was available which were ultimately reported at follow-up to be true positives, and the non-pathological scans which were reported at follow-up to be false negatives

Data detailing subsequent treatment decisions was available for 27/65 (42%) of the dPET/CT cohort and 38/65 (58%) of the aPET/CT cohort. Likewise, confirmatory imaging, post therapy PSA (or a combination of both), or histological verification was available for *n* = 27 of the dPET/CT cohort and *n* = 32 of the aPET/CT cohort. Scans where a majority (≥ 3 readers) agreed that at least one pathological (PSMA-RADS 4-5) lesion was present (i.e. PSMA-scan positive) were compared against the available confirmatory data by the composite standard. For dPET/CT, all 27 patients for whom confirmatory data were available were confirmed as true positives with no false positives and no false negatives. For aPET/CT, of 32 patients in whom follow-up data were available, 27 had a true positive and 5 had a false negative. As such, the true-positive rate for dPET/CT was significantly higher compared to aPET/CT (1.0 vs. 0.84 respectively, *p* < 0.0001), as shown in Fig. 5.

### Frequency of discrepant reading, interrater agreement, and reliability

**Fig. 6:**
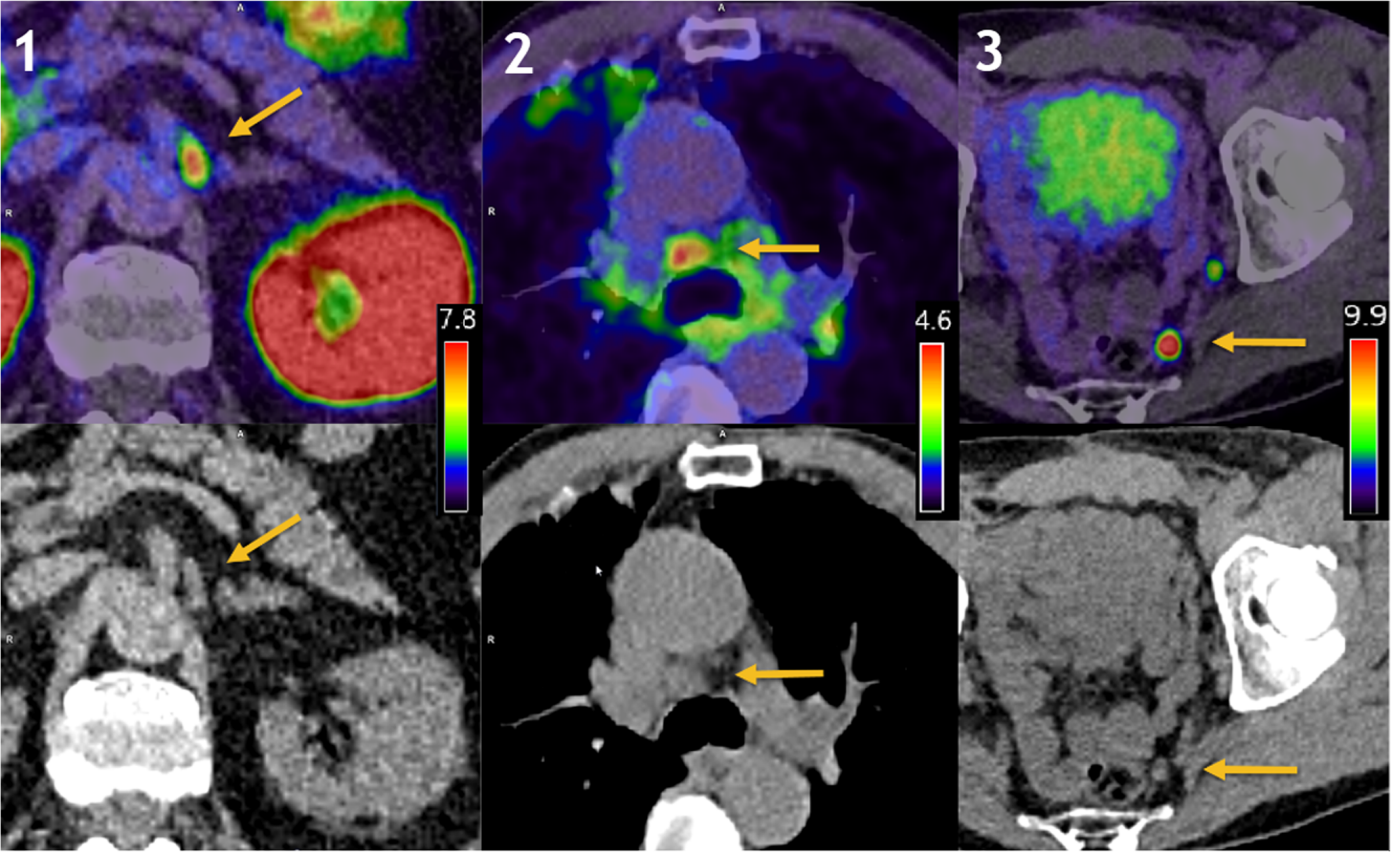
Example images showing a benign ganglion with intensive PSMA-uptake (1, leftmost), equivocal uptake in a mediastinal lymph node (2, middle), and clearly pathological uptake (3, rightmost) in a pelvic lymph node. Top row fusion of PET and CT, bottom row. The appropriate window levels affording the best appreciation of the lesion are shown in the legend for each panel

Scans which were unanimously rated by all four readers as pathological and negative were noted, and scans where one or more readers differed in opinion were noted as discrepant. A small, albeit non-significant difference in the frequency of discrepant scans was noted between dPET/CT and aPET/CT (20% vs. 16% respectively, *p* = 0.18). Interrater reliability for the number of pathological lesions detected between each reader for both scanners was excellent (Cronbach’s *α* = 0.923 dPET/CT; *α* = 0.948 aPET/CT). Interrater agreement for the number of pathological lesions was substantial for dPET/CT and almost perfect for aPET/CT (Krippendorf’s *α* = 0.701 dPET/CT; 0.802 aPET/CT). Example PET/CT images are shown in Fig. 6 showing benign uptake in a ganglion (PSMA-RADS 1B), equivocal uptake in a mediastinal lymph node (PSMA-RADS 3C), and a clearly pathological lymph node (PSMA-RADS 5).

## Discussion

We present here a retrospective matched-pair cohort analysis for a matched pair of patients undergoing dPET/CT or aPET/CT in the context of recurrent PC following initial radical prostatectomy, or radical prostatectomy combined with radiotherapy. In keeping with previous publications, our blinded read by four independent physicians reveals greater numbers of lesions (benign, equivocal, and pathological) for dPET/CT compared with aPET/CT (*p* < 0.001). Our analysis showed that dPET/CT detected no significantly increased rate of non-specific lesions (dPET/CT 61.8%, aPET/CT 57% *p* = 0.4) and a slightly lower (non-significant) rate of equivocal lesions (dPET/CT 11.5%, aPET/CT 13.7%, *p* = 0.4). This implies that, despite increased sensitivity, the readers were not disproportionately confronted with diagnostic conundrums, confirming previous reports of improved diagnostic confidence (albeit based on a subjective 5-point scale) in dPET/CT by Nguyen et al [4].

Yin et al showed, using the PSMA-RADS classification system, that a significant proportion of equivocal lesions can show evidence of malignancy at follow-up [27], highlighting their potential clinical significance. Given that a number of studies report only binary pathological/benign scales and that diagnostic pitfalls are reported in increased numbers for ^18^F-labelled radioligands such as [^18^F]PSMA-1007 of [^18^F]DCFPyl, we consider that in our finding, no increased rate of equivocal lesions at dPET/CT is of potential importance [28, 36].

This higher rate of lesion detection translated into a higher rate of patient-based sensitivity (i.e. increased rate of pathological scans), confirming previous preliminary studies reporting improved lesion detection by such systems [4, 5, 7]. Given the impact that pathological PET/CT has on future treatment planning, particularly at low PSA values, the finding that the patient-based sensitivity is significantly higher is of potential clinical significance [37].

The number of scans reported as pathological was higher in dPET/CT for each reader. This improved patient-based sensitivity for dPET/CT finds explanation in the physical characteristics of the scanner; both clinical observations and phantom studies demonstrate a higher sensitivity and improved tumour-to-background ratio for dPET/CT, which we posit to also improve visual discrimination of PSMA-avidity [7]. This raises important questions for the design of future studies, given the limited comparability of not just semi-quantitative data between dPET/CT and aPET/CT (such as SUV, which is previously reported to differ significantly [6]) but, as we show here, also for qualitative measures such as the interpretation and classification of lesions between systems.

Using a composite standard of follow-up, we investigated the diagnostic accuracy (at a patient-based level) for scans reported as “pathological” or “non-pathological” (i.e. where a majority of readers report at least one lesion classified as pathological). For dPET/CT, we find no examples of false-positive or false-negative scans, giving a true-positive scan rate of 100%. However, for aPET/CT, of the 32 patients for whom follow-up details were available, only 27 had true-positive scans, with 5 scans incorrectly reported by our readers as negative (with a confirmatory composite standard of truth). The lower rate of equivocal findings, coupled with an improved true-positive rate, implies that although readers were confronted with more lesions at dPET/CT, they were better able to classify them and that this classification was more accurate. This points toward an important advantage of dPET/CT in our study.

The rate of interrater reliability was excellent for both scanners (Cronbach’s *α* = 0.923 dPET/CT; *α* = 0.948 aPET/CT), with no increased rate of scans where one or more readers differed in opinion. Agreement was substantial (dPET/CT Krippendorf’s *α* = 10.701) to almost perfect (aPET/CT Krippendorf’s *α = *0.802), confirming previous estimates of agreement [12, 36, 38]. In keeping with previous studies [36, 38], we do note variation in reader experience: the most junior readers reported significantly higher rates of benign lesions for both dPET/CT compared to aPET/CT and the most junior reader revealed a slightly higher rate of equivocal lesions in dPET/CT, suggesting a learning curve is encountered with both dPET/CT and aPET/CT. Further studies are required to confirm the minimum number of scans required.

In addition to those already noted, we note several limitations to our study. Ideally, these data would be confirmed by an intra-patient analysis, although our matched-pair cohort was as closely matched for clinical parameters as possible with no statistically significant differences for age, Gleason score, or PSA value and matching TNM stage. Radiopharmaceutical doses were given according to the same body-weight-adjusted target. Although intra-individual comparisons are preferable [6], such a design does not lend itself to studies using ^68^Ga radiotracers, where the short half-life limits the value of comparisons at differing time-points [39, 40], particularly where a steady-state regime cannot be assumed [18]. We cannot entirely rule out selection bias in this retrospective study, although the date ranges for each cohort were non-overlapping, ruling out scanner choice as a potential confounder. Further studies with larger patient cohorts are required to confirm our findings. However, a power analysis confirmed that the sample size chosen was appropriate. In common with most studies in recurrent PC, we cannot provide lesion-based histological verification [32]. In mitigation, clinical follow-up was performed for all patients, with a follow-up rate comparable to that of previously published cohort studies [25] and using a composite standard of truth as previously published [9]. For all patients where data was available, a composite follow-up of post-treatment PSA decline or concordant imaging (MRI) was available. No false-positive findings were noted. When rating scans, all readers were provided with written details of the PSMA-RADS classification system [31] and details regarding known pitfalls and causes of benign radiotracer uptake (e.g. dorsal root ganglia, benign bone disease) were provided [15–17, 19, 41], further minimising the risk of false positives. Finally, we are cognisant of the differences in reconstruction parameters on image quality, particularly voxel size [42, 43] and instead we demonstrate the best-possible performance of the systems under real-world clinical conditions [44].

## Conclusion

This study provides data for the influence of dPET/CT on interrater reliability and diagnostic certainty for [^68^Ga]Ga-PSMA-11 for the staging of recurrent PC. Our results demonstrate a higher detection rate for pathological lesions and higher patient-based sensitivity for dPET/CT when compared with aPET/CT. We find this to be commensurate with the known higher sensitivity demonstrated for dPET/CT systems. This improved sensitivity was coupled with improved diagnostic performance, as shown by the higher true-positive rate, and was not at the cost of reduced interrater reliability or diagnostic certainty.

## Abbreviations

2-[^18^F]FDG: 2-Deoxy-2-[18F]fluoro-D-glucose
aPET/CT: Analogue PET/CT
BGO: Bismuth germinate
CT: Computed tomography
dPET/CT: Digital PET/CT
GS: Gleason score
MRI: Magnetic resonance imaging
OP: Operation, in this context, post-prostatectomy management of the primary prostate cancer
PC: Prostate cancer
PET: Positron emission tomography
PSA: Prostate-specific antigen
PSMA: Prostate-specific membrane antigen
PSMA-RADS: Prostate-specific membrane antigen reporting and data system
RT: Radiotherapy, in the context of this study, post-radiotherapy management of the primary prostate cancer
STARD: Standards for reporting diagnostic accuracy studies
TNM: Tumour, nodes and metastasis (American Joint Committee on Cancer staging system)
UICC: Union for International Cancer Control

## Declarations

### Guarantor

The scientific guarantor of this publication is Dr. Ian Alberts

### Statistics and biometry

No complex statistical methods were necessary for this paper.

### Study subjects or cohorts overlap

Some study subjects or cohorts have been previously reported in Alberts I, et al *Eur J Nucl Med Mol Imaging* 2020 Mar;47(3):614-623. doi: 10.1007/s00259-019-04630-y. Epub 2019 Dec 2

### Methodology

• Retrospective

• Observational

• Performed at one institution
